# Myelin basic protein and ischemia modified albumin levels in acute ischemic stroke cases

**DOI:** 10.12669/pjms.315.7702

**Published:** 2015

**Authors:** Serdar Can, Okhan Akdur, Ahmet Yildirim, Gurhan Adam, Dilek Ulker Cakir, Handan Isin Ozisik Karaman

**Affiliations:** 1 Serdar Can, MD. Departments of Emergency Medicine, Canakkale Onsekiz Mart University Faculty of Medicine, Canakkale, Turkey; 2 Okhan Akdur, MD. Associate Professor, Departments of Emergency Medicine, Canakkale Onsekiz Mart University Faculty of Medicine, Canakkale, Turkey; 3 Ahmet Yildirim, MD. Assistant Professor, Departments of Emergency Medicine, Canakkale Onsekiz Mart University Faculty of Medicine, Canakkale, Turkey; 4 Gurhan Adam, MD. Assistant Professor, Departments of Radiology, Canakkale Onsekiz Mart University Faculty of Medicine, Canakkale, Turkey; 5 Dilek Ulker Cakir, MD. Associate Professor, Departments of Biochemistry, Canakkale Onsekiz Mart University Faculty of Medicine, Canakkale, Turkey; 6 Handan Isin Ozisik Karaman, MD. Professor, Departments of Neurology, Canakkale Onsekiz Mart University Faculty of Medicine, Canakkale, Turkey

**Keywords:** Acute stroke, Cerebral Ischemia, Ischemia-modified serum albumin

## Abstract

**Objective:**

To investigate early diagnostic effects of serum myelin basic protein (MBP) and ischemic modified albumin (IMA) levels in patients with ischemic stroke.

**Methods:**

Fifty patients who presented to an emergency service with acute ischemic stroke between June 2013 to March 2014 were evaluated with the National Institute of Health Stroke Scale (NIHSS) and diffusion-weighted magnetic resonance imaging (MRI). Thirty four healthy cases were included as control group. All patients’ serum IMA and MBP level were assessed.

**Results:**

Mean IMA value was 0.52±0.25 cases with acute ischemic stroke and serum IMA levels were significantly higher than the control group (p<0.01). No statistical significance was observed between acute ischemic stroke group and control group related to the MBP serum levels (P>0.05). Statistically significant correlation was detected between the volumes of diffusion restriction on MRI and NIHSS score (P=0.002, r=0.43) and IMA (P=0.015, r=0.344) levels.

**Conclusions:**

We have found that serum IMA levels are elevated in acute ischemic stroke cases and these levels are correlated with the ischemic tissue volume. MBP levels do not increase in early period of stroke cases.

## INTRODUCTION

Acute ischemic stroke is caused by sudden interruption of cerebral blood flow. Morbidity and mortality of acute ischemic stroke cases are still high besides the developments in the treatment. Especially early diagnosis is required for an effective reperfusion therapy.^1^ Therefore, minimization of the time passed between the correct diagnosis and the beginning of the symptoms is very important. Diagnostic evaluation of suspected acute ischemic stroke cases in emergency services requires technical equipment and time consuming methods such as brain computerized tomography (CT) or magnetic resonance imaging (MRI) that could be performed by trained personnel.^2^

Thrombolytic treatment which could decrease mortality and improve quality of life is carried out in the first 3 hours of acute ischemic stroke cases. It is stated that thrombolytic treatment is more beneficial especially in cases with low infarct volume in diffusion-weighed imaging and National Institute of Health Stroke Scale (NIHSS).^3^. Therefore, early diagnosis and calculation of infarct volume are the parameters used for evaluation of eligibility for thrombolytic treatment.^4^

CT has low sensitivity and specificity in early diagnosis of acute ischemic stroke. MRI is not available in all medical facilities managing emergency cases. For this reason, it is thought that serum biomarkers could be used for early diagnosis of acute ischemic stroke and estimation of infarct volume. It is stated that some neuronal and glial proteins could be markers of ischemic stroke.^5^-^7^

Myelin basic protein (MBP) is a protein that is attaching myelin sheath to oligodendrocytes and its serum levels are stated to increase in early period after acute ischemic stroke.^8^,^9^ Ischemic modified albumin (IMA) is a molecule that is produced by decreased albumin metal-binding capacity in case of acute ischemic stroke. It is recently accepted as a sensitive biochemical marker for myocardial ischemia, muscle ischemia, pulmonary embolism and mesenteric ischemia.^10^,^11^

We have evaluated MBP and IMA serum markers, which we thought will provide possibility of early diagnosis and referral to imaging unites early for reperfusion treatments in acute ischemic stroke patients. We have investigated the efficacy of these parameters in early diagnosis and establishment of infarct volume by calculating MBP and IMA serum levels in acute ischemic stroke patients and ischemic region volumes in diffusion-weighed MRI images.

## METHODS

### Study Design

This prospective study was conducted in Canakkale Onsekiz Mart University Faculty of Medicine Research and Training Hospital (COMURTH) Emergency Medicine Department. COMURTH is a university health care institution located in North-east of Turkey, providing service to a population of around 500.000. This study was conducted between June 2013 to March 2014 with 50 acute ischemic stroke patients (stroke group) that were admitted to the Emergency Medicine Department and 34 healthy cases (control group) from general outpatient department with informed consent of patients and the approval of Canakkale Onsekiz Mart University Faculty of Medicine Ethical Committee.

Cases aged over 18 years, admitted to emergency department in the first 12 hours after the acute ischemic stroke symptoms started and detected to have acute ischemic stroke findings on diffusion-weighed MRI for the first time were included in the study. Clinical findings of acute ischemic stroke defined by World Health Organization were present in all cases.^12^ Venous blood samples were collected during neurological examinations performed at first admittance for measurement of MBP and IMA levels.

Age, gender, previous medical history, Glasgow Come Scale (GCS), NIHSS and ischemic region volume on diffusion-weighed MRI were recorded for all cases.

Cases with a previous diagnosis of ischemic stroke, deep vein thrombosis and arterial occlusion could not be excluded, intracranial pathology, impaired kidney and liver functions, infection symptoms, immunological diseases, malignancy and pregnancy, myocardial infarction and pulmonary embolism diagnosis within last one year, abnormal serum albumin levels, that are not possible to perform MRI and diagnosed with transient ischemic stroke were excluded from the study. Same exclusion criteria had been used for the control group.

### Evaluation

Acute ischemic stroke cases were evaluated with physical examination, neurological examination, GCS and NIHSS scores. Level of consciousness was graded as mild (15), moderate (8-14) and severe (3-7) with GCS. Stroke severity was evaluated with NIHSS at the admission. Mild<8; moderate 8-16; severe>17.^13^ Blood samples were collected for IMA, MBP serum level measurements from stroke and control groups. All the patients had standard stroke management in accordance with guidelines of the neurology unit of COMURTH.

### Biochemistry

Blood samples were centrifuged at 800-1000 Rpm for 10 minutes and stored in eppendorf tubes at -80°C for biochemical tests. Enzyme-linked Immunabsorbent Assay Kit for Myelin Basic Protein (Uscn Life Science Inc. E90539 Hu 96 Tests) was used for MBP level measurement. MBP level measurement was performed with ELISA method and measured as pg/mL.

Colorimetric method was used for serum IMA level measurements in our study. Serum samples were studied with the method defined by Bar-Or et al.^14^ 200 μL serum is added to 0.1% (w/v) of 50 μL cobalt chloridine solution in this method. Albumin cobalt binding reaction was waited to happen at sufficient level by gentle mixing for 10 minutes. Fifty μL dithiothreitol (DDT) (1.5 mg/mL H_2_O) colouring agent was added afterwards. 1.0 mL 0.9% NaCl was added to stop the reaction after two minutes. Afterwards, colour changing was measured as 470 nm spectrophotometry. Measurement results were reported in Absorbance Units (ABSU).

### Imaging

Brain computerized tomography scans were performed to exclude intracerebral hemorrhage and other pathology that could imitate ischemic stroke at the admittance. 1,5-T MRI device (GE Signa HDxt Series, USA) was used to view the ischemic region. All imaging techniques were commented by a radiologist for findings of ischemic stroke and ischemic region volume measurements were performed. Images were reformatted from volumetric ADC maps acquired by using AW volume share 2 software and GE work unit. Restriction areas were marked by Quick Point program in all sections in axial, coronal, and sagittal planes and the volume of diffusion restriction area was calculated automatically in cm^3^.

### Statistical Analysis

Data analysis was performed using Statistical Package for the Social Sciences (SPSS) ® version 19.0. Categorical data are summarized as frequencies and percentages while continuous data are described as mean values ± standard deviation (SD). We used the Kolmogorov-Smirnov test to assess the normality of variables. Comparison of group differences in categorical variables was achieved using X^2^ test, while the Student’s t-test and Mann Whitney U test were used to compare means of continuous normally distributed and non-normally distributed variables respectively. Statistical significance was set at p<0.05. Correlations among laboratory parameters and MRI volume were analysed using Spearman’s rank correlation test. Values below p<0.01 was accepted as statistically significant.

## RESULTS

Fifty ischemic stroke cases and 34 healthy control cases were included in the study. Mean age of the stroke cases included in the study was 68.14±14 years and 52% of these cases were males. Demographical and clinical characteristics of the cases are shown in Table-I.

**Table-I:** Demographic and clinical characteristics of patients who had or did not have stroke.

	Stroke	Control	P value
Patients number	50	34	-
Age (years)	68.1±14	58±13.1	P>0.05
Sex (Male)	26	18	P>0.05
Glasgow coma scale	14.5±1.2	-	-
NIHSS	5.5±4.3	-	-
MBP (pg/mL)	82.4±63.9	73±47.5	P>0.05
IMA (ABSU)	0.52±0.25	0.35±0,04	P<0.01

Data reported as number (%) or mean ± standard deviation. Statistical significance was defined as P<0.05.

Mean IMA value was 0.52±0.25 ABSU among cases with acute ischemic stroke. Serum IMA levels of acute ischemic stroke cases was significantly higher than the control group (p<0.01). No statistical significance was observed between acute ischemic stroke group and control group related to the MBP serum levels (p>0.05) (Table-I).

Mean NIHSS score of stroke group was 5.5±4.3 and mean GCS was 14.5±1.2. No statistical correlation was detected between serum MBP (p>0.05, r=-0.07) and IMA (p>0.05, r=0.25) levels and NIHSS scores of stroke cases.

No statistically significant difference was observed between MBP and IMA levels of stroke cases with mild and moderate GCS (Table-II). Mean diffusion restriction area volume in MRI of acute ischemic stroke cases was 11.0±116 cm^3^, minimum volume was 0.058 cm^3 and^ maximum was measured as 61.3 cm^3^.

**Table-II:** Evaluation of GKS, MBP and IMA levels in acute ischemic stroke patients.

Parameter	Glasgow Coma Scale						
	Mild (15)		Moderate (7 to 14)		Severe (3 to7)		
	No. of Patients	Mean±SD	No. of Patients	Mean±SD	No. of Patients	Mean±SD	P value
MBP (pg/mL)	39 (78)	82.3±61	11 (22)	82.9±76.7	-	-	0.61
IMA (ABSU)	39 (78)	0.5±0.2	11 (22)	0.6±0.2	-	-	0.16

Data reported as number (%) or mean ± standard deviation. Statistical significance was defined as P<0.05.

**Fig.1:**
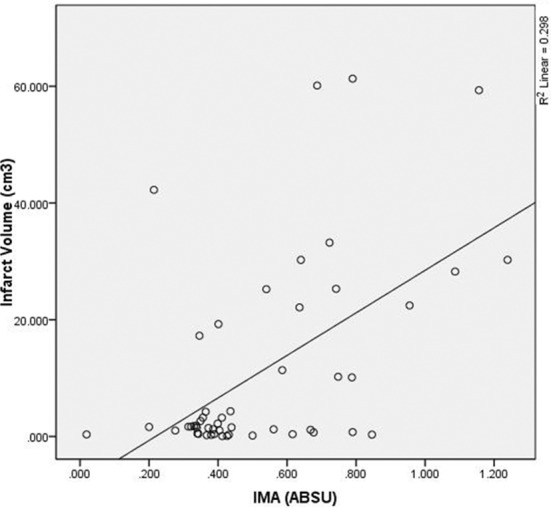
Correlation between the plasma IMA levels and the infarct volume.

No statistically significant correlation was detected between the volumes of diffusion restriction on MRI and serum MBP (p>0.05, r=0.16) levels of stroke cases. Statistically significant correlation was detected between the volumes of diffusion restriction on MRI and NIHSS score (p<0.01, r=0.43) and IMA (p<0.01, r=0.344) levels (Table-III) (Fig.1).

**Table-III:** Correlation between IMA, MBP, NIHSS and infarct volumes.

		NIHSS	MBP (pg/ml)	IMA (ABSU)
Mean±SD		5.54±4.3	82.5±64.0	0.52±0.25
Infarct volume 11.0±16.7 cm^3^	P	0.002^*^	0.26	0.015^*^
	R	0.43	0.16	0.34
	N	50	50	50

*Correlation is significant at the 0.05 level (2-tailed).

## DISCUSSION

Acute ischemic stroke is still an important reason of mortality and morbidity besides the developments achieved in treatment. The most important obstacle for suspected cases of acute ischemic stroke is the failure of very quick diagnostic evaluation. And this may cause delayed diagnosis and treatment. MRI which is used for diagnostic imaging is a method requiring trained personnel and technical facilities and is not available in many health care centers. Thus, we think that diagnostic serum markers such as BMP and IMA will provide important clinical benefits for the diagnosis of acute ischemic stroke.

The most important result of this study was that IMA levels of acute ischemic stroke cases were higher than the control group. For this reason, we think that it could be used for evaluation and referral to imaging units for diagnosis of suspected cases of acute ischemic stroke admitted to emergency services. IMA is accepted as a nonspecific marker of tissue ischemia.^15^ Oxidative stress during acute stroke combined production of reactive oxygen radicals and free oxygen radicals contribute to production of IMA through damaged blood brain barrier.^15^,^16^ It was reported that IMA levels are higher in ischemic stroke cases than the healthy individuals in other studies.^15^,^17^ However, another important data that we have acquired from our study is that there was a positive correlation between infarct volumes calculated from diffusion MRI images and IMA levels. Thus, it could be thought that it also might be a prognostic marker in addition to diagnosis. However, elevated IMA levels were not correlated with NIHSS and GCS scores which are thought to show severity of stroke. In another study it is reported that IMA levels are higher in cases of intracerebral hemorrhage, infarction and transient ischemic attack, epileptic attacks than the healthy individuals. In that study it was shown that there is a correlation between elevated IMA levels and NIHSS score.^18^

Another important finding is that we have evaluated serum BMP levels of stroke cases at the time of admission. MBP is a structural component of CNS myelin and it is stated to increase in case of neurodegeneration.^19^ However, we have not detected an increase of BMP levels of stroke cases. No correlation was found between both NIHSS and GCS scores and MBP levels. Studies about MBP reported that it is not a good plasma marker in first 6 hours of stroke. It is reported that the duration of passage to the circulation is long and it is increased in the serum in subacute period.^19^ MBP is more elevated especially in cases of severe stroke cases than the mid stroke cases.^20^ According to our results as well, MBP levels were not increased in early period of acute ischemic stroke. MBP is revealed during myelination of nerves, so in demyelinization MBP level may increased.

Markers of tissue damage in brain are released to the blood from glia, myelin and neurons following a stroke. These markers might contribute to easier and cheaper referral of cases to imaging techniques. The best marker is thought to be correlated with the volume of damaged tissue. But, volume measurement is quite difficult. Volume measurements could be evaluated differently in MRI and CT scanning, whether measurement is done manually or automatically and according to the time of the imaging (acute, subacute).^19^,^21^

We have calculated the volume diffusion restriction automatically with volume software in cm^3^ in our study. We have found that volumes measured were correlated with the IMA levels and NIHSS scores. However, ischemic tissue volume might not be a data showing the last stage of clinical outcome in stroke patients. Therefore, studies with longer duration of follow-up should be conducted.

### Limitations of the study

It was conducted with a small group of stroke patients. Diffusion-weighed magnetic resonance images obtained at the admission of the patients to the emergency service was used for infarction volume measurements. It should be taken into consideration that infarction dimensions could increase. Therefore, it will be more appropriate if the number of the patients is increased and MRI images that are obtained during follow-up are also used in addition to those obtained at admission.

In conclusion, we have found that serum IMA levels are elevated in acute ischemic stroke cases and these levels are correlated with the ischemic tissue volume. MBP levels do not increase in early period of stroke cases.
